# Quorum sensing as a potential target for increased production of rhamnolipid biosurfactant in *Burkholderia thailandensis* E264

**DOI:** 10.1007/s00253-019-09942-5

**Published:** 2019-06-21

**Authors:** Irorere U. Victor, Michal Kwiencien, Lakshmi Tripathi, Diego Cobice, Stephen McClean, Roger Marchant, Ibrahim M. Banat

**Affiliations:** 1 School of Biomedical Sciences, Faculty of Life and Health Sciences, Ulster University, Coleraine, BT52 1SA Northern Ireland, UK; 2 Centre of Polymer and Carbon Materials, Polish Academy of Sciences, 34, M. Curie-Skłodowska St, 41-819 Zabrze, Poland

**Keywords:** Rhamnolipid, Quorum sensing, *Burkholderia thailandensis*, Wild type, Triple mutant

## Abstract

*Burkholderia thailandensis* E264 is a potential non-pathogenic substitute for producing rhamnolipid biosurfactant, replacing the pathogenic *Pseudomonas aeruginosa*. However, it has low rhamnolipid production and longer fermentation time. We have earlier suggested that media supplementation with exogenous quorum sensing (QS) molecules could lead to early onset of biosynthesis and increased rhamnolipid yield. Here, we assessed the effect of single, double or triple mutations in the various QS systems of *B. thailandensis* on rhamnolipid production, with the view to see which system(s) have the most impact on rhamnolipid yield and subsequently use the QS molecule to potentially increase yield in the wild-type *B. thailandensis*. The triple mutant strain had a rhamnolipid yield of 4.46 ± 0.345 g/l at 240 h of fermentation which was significantly higher than that of the wild type (0.94 ± 0.06 g/l), an unexpected outcome. To gain more insight as to how this might occur, we studied substrate metabolism and energy storage in the form of polyhydroxyalkanoate (PHA) by both the triple mutant and the wild type. We observed increased glycerol metabolism and reduced PHA production in the triple mutant compared with the wild type. Glycerol concentration at 240 h and maximum PHA productivity (g/gDCB) were 8.76 g/l or 16.19 g/l and 21.80% or 31.4% in either the triple mutant or the wild type respectively. Complementation of the triple-mutant cultures with exogenous QS molecules restored rhamnolipid production to similar levels as the wild type. QS therefore is a potential target for increased rhamnolipid production in *B. thailandensis*.

**Electronic supplementary material:**

The online version of this article (10.1007/s00253-019-09942-5) contains supplementary material, which is available to authorized users.

## Introduction

The term quorum sensing (QS) describes the cell-to-cell communication mechanism/s in bacteria, which produces coordinated behaviours within a bacterial population upon the establishment of a quorum. This is achieved through the production, sensing and response to chemical signalling molecules known as pheromones, quorumones or autoinducers (AIs) (Dusane et al. 2010; Rutherford and Bassler 2012; Williams 2007). Bacteria that use this system produce and release these signal molecules into the environment, the accumulation of which increases with increasing bacterial population. Consequently, a threshold concentration is reached, leading to the interaction of the signal molecules with cognate receptors forming a signal transduction cascade that ultimately results in the expression or repression of QS regulated genes (Papenfort and Bassler 2016; Williams 2007).

QS-regulated processes are usually those that are costly or ineffective when carried out by a single bacterium, but become effective when carried out by a population of bacteria. This phenomenon has been described as one that enables a bacterial population to act as a multicellular organism and obtain benefits which are unachievable should they act alone (Bassler and Losick 2006). Well-known QS-regulated processes include bioluminescence, biofilm formation and secretion of virulence factors (Bassler and Losick 2006; Papenfort and Bassler 2016). Specifically, QS has been implicated in the regulation of various bacterial physiological processes including antibiotic resistance, antibiotic synthesis, production of exopolysaccharide and rhamnolipid synthesis (Bjarnsholt et al. 2005; Duerkop et al. 2009; Marketon et al. 2003; Nickzad and Déziel 2016; Nickzad et al. 2015).

In the last three decades, the effect of quorum sensing on rhamnolipid production has been extensively studied, particularly in strains of *Pseudomonas aeruginosa* (Ahmed et al. 2019). Three different autoinducers regulating rhamnolipid production have been identified in *P. aeruginosa*: *Pseudomonas* autoinducer 1 or PAI-1 (N-(3-oxododecanoyl-homoserine lactones or 3-oxo-C12-HSLs), *Pseudomonas* autoinducer 2 or PAI-2 (N-butyryl-homoserine lactones or C4-HSLs) and *Pseudomonas* Quinolone Signal or PQS (2-heptyl-3-hydroxy-4-quinolone) (Pearson et al. 1994; Pearson et al. 1995; Pesci et al. 1999). PAI-1 synthesis is under the control of the LasI–LasR QS system while the rhlI–rhlR QS system controls the synthesis of PAI-2; these 2 systems are known to directly control rhamnolipid biosynthesis in *P. aeruginosa* (Dusane et al. 2010). Although the PQS autoinducer does not belong to a specific QS system, it regulates rhamnolipid production by either direct or indirect regulation of the synthesis of C_4_-HSL or PAI-2 belonging to the rhlI–rhlR QS system (Dusane et al. 2010; McKnight et al. 2000).

Single or multiple mutations in genes responsible for the synthesis of the different autoinducers have been shown to result in the decrease or absence of rhamnolipid synthesis in *P. aeruginosa* (Pearson et al. 1997). Furthermore, the addition of either exogenous synthetic autoinducers or spent media containing high concentrations of autoinducers has been reported to result in increased rhamnolipid biosynthesis in *P. aeruginosa* (Nakata et al. 1998; Ochsner et al. 1994).

These results do show a direct correlation between rhamnolipid production and quorum sensing. It also suggests that quorum sensing is a major target for increasing rhamnolipid productivity in microbial strains, particularly in the early growth stages of *Burkholderia thailandensis*, as we have previously suggested (Irorere et al. 2018). However, very little is known about the effect of quorum sensing on rhamnolipid biosynthesis in *Burkholderia species*. Recent studies on *B. glumae* have shown that the mutant strain of *B. glumae tofl*^*−*^, deficient in the synthesis of N-3-octanoyl-homoserine lactone (C_8_-HSL), has reduced rhamnolipid production. This shows that rhamnolipid production is positively regulated by QS in *B. glumae* (Nickzad et al. 2015).

However, to the best of our knowledge, there are no available reports on the effect of quorum sensing on rhamnolipid production in *B. thailandensis*. In 2009, Chandler et al. characterised the QS systems in *B. thailandensis*. They confirmed that 3 QS systems were present in *B. thailandensis*, similar to those in *B. pseudomallei* but different from *B. glumae* which has a single QS system. The 3 QS systems in *B. thailandensis* comprise 3 pairs of synthase/receptors and 2 orphan receptors with no known synthase (Chandler et al. 2009; Ulrich et al. 2004). The quorum sensing system one or QS-1 in *B. thailandensis* comprises *btaI1* and *btaR1* genes, coding for the synthesis of the synthase and transcriptional regulator BtaI1 and BtaR1 respectively, which respond to and produce the autoinducer N-octanoyl homoserine lactone (C_8_-HSL), while the QS-2 system consists of the *btaI2* and *btaR2* genes coding for the BtaI2 synthase and the BtaR2 transcriptional regulator that responds to and regulates the synthesis of both N-3-hydroxyldecanoyl homoserine lactones (3OHC10-HSL) and N-3-hydroxyloctanoyl homoserine lactone (3OHC_8_-HSL). Finally, the QS-3 system is made up of the *btaI3* and *btaR3* genes coding for BtaI3 and BtaR3 synthase and transcriptional regulator respectively, which respond to and regulate the biosynthesis of N-3-hydroxyloctanoyl homoserine lactone (3OHC_8_-HSL) (Chandler et al. 2009; Le Guillouzer et al. 2017; Tseng et al. 2016).

This present study is focused on the effect of quorum sensing mutations on rhamnolipid production in *B. thailandensis* E264. We report that unlike *P. aeruginosa* or *B. glumae*, quorum sensing–deficient mutants of *B. thailandensis* E264 have increased rhamnolipid production compared with the wild type.

## Materials and methods

### Microbial strains

Rhamnolipid production was carried out by *Burkholderia thailandensis* ATCC 700388. A list of all mutant strains used in this study is presented in Table S1. Mutant strains were kindly provided by Dr. Josephine Chandler from the University of Kansas and the University of Washington, USA. The method used for making quorum sensing mutant strains is a markerless allelic exchange method and is fully described by Chandler et al. (2009)). Upon receipt, strains were immediately sub-cultured in nutrient broth for 24 h, after which cultures were transferred to cryovials containing 50% glycerol at a ratio of 1:1 and kept at − 80 °C before use. Prior to use, fresh stocks were taken from the freezer and streaked on nutrient agar (Sigma-Aldrich, UK) plates, incubated for 24 h at 30 °C. Fresh nutrient agar plates were prepared every 2 weeks, and care was taken to avoid cross contamination.

### Rhamnolipid production

Initial rhamnolipid production was carried out for all mutant strains and the wild type, including single (S*ΔbtaI1*, S*ΔbtaI2* and S*ΔbtaI3*) double (D*ΔbtaI2ΔbtaI3*) and triple (T*ΔbtaI1ΔbtaI2ΔbtaI3*) mutants. Single colonies of respective strains were used to prepare a starter culture in nutrient broth (Sigma-Aldrich, UK), grown at 30 °C and 200 rpm, to an OD_600_ of approximately 2.3. These were used to inoculate the rhamnolipid production media (nutrient broth, with 4% (*w/v*) glycerol) in a 1-l Erlenmeyer flask at a 10% inoculum ratio. For rhamnolipid production, inoculated flasks were incubated at 30 °C and 200 rpm for 240 h with samples collected regularly to quantify rhamnolipid production, assess growth, measure surface tension and determine glycerol concentration. All experiments were carried out in biological triplicates, with additional flasks inoculated to quantify rhamnolipid concentration at different times of fermentation.

A complementation study was carried out on the triple mutant strain only (*TΔbtaI1ΔbtaI2ΔbtaI3*), with the wild type used as control. Two different complementation analyses were carried out. The first involves growing the wild-type *B. thailandensis* E264 in nutrient broth for 24 h at 30 °C and 200 rpm. Cells were removed by aseptic centrifugation, and the supernatant was sterilised by filtration with a 0.25-μm syringe filter (Sartorius Minisart Plus). No growth was observed on nutrient agar plates inoculated by spread plating 100 μl of the filtered spent medium. Fifty millilitres of the filtered spent medium was added to a 40-ml sterile fresh nutrient broth containing 4 g glycerol; this was inoculated with a 10-ml starter culture, giving a 4% (*w/v*) carbon source concentration.

The second complementation involved supplementation of the fermentation nutrient broth (containing 4% glycerol) with 4 μM each of synthetic 3OHC_10_-HSLs, 3OHC_8_-HSLs and C_8_-HSLs (Cambridge Bioscience, UK). The medium was inoculated with 10% (*v/v*) inoculum concentration. All complementation studies were carried out in triplicate with the wild-type strain used as a control.

### Rhamnolipid extraction and purification

To quantify rhamnolipid produced during fermentation, 30 ml of fermentation broth was taken and centrifuged for 15 min at 13,000 *g*. Biomasses were separated from the supernatant by transferring the supernatant to new 50-ml tubes, and rhamnolipid extraction was carried out by acid precipitation and solvent extraction as previously described (Irorere et al. 2018; Smyth et al. 2014). This involves reducing the pH of the supernatant to 2.0 followed by three subsequent extractions with ethyl acetate. Anhydrous MgSO_4_ was used to remove the residual aqueous phase from the organic phase containing rhamnolipid. This was followed by filtration, rotary evaporation of solvent, transfer of rhamnolipid to vials and removal of any residual solvent with nitrogen gas. The weight of the extracted rhamnolipid was determined gravimetrically.

Extracted rhamnolipid were purified prior to characterisation by solid phase extraction (SPE), using Strata SI-1 Silica (55-μm, 70-A) Giga tubes (Phenomenex) as previously described (Irorere et al. 2018).

### HPLC-MS analysis of rhamnolipid

After purification, the determination of the relative proportions of rhamnolipid congeners present in each sample was carried out by HPLC-MS. The system used consisted of a classic LCQ ion trap mass spectrometer (Thermo Finnigan, UK) coupled to a SpectraSYSTEM LC P4000 (Thermo Finnigan, UK) high-performance liquid chromatography system, equipped with a 150 × 4.6-mm Kinetex 5-μM F5 100-Å (Phenomenex) reversed-phase LC column. The mass spectrometer was operated at a flow rate of 0.8 ml/min in the negative ionisation mode with ion spray voltage of 4500 V, capillary voltage of − 25 V and capillary temperature of 250 °C. The chromatography was conducted in gradient mode, using water and acetonitrile as mobile phases A and B respectively and a sample injection volume of 10 μl. The gradient profile used is shown in Table S2.

For quantification, purified rhamnolipid was used as a standard to create a calibration curve (Fig. S1). Quantification was performed in single ion recording (SIR) mode using normalised internal standard (stearic acid). Different pseudomolecular ions representing monorhamnolipid and dirhamnolipid were initially used as quantifier ions, to study their linearity with increasing rhamnolipid concentrations. The pseudomolecular ions 733 *m/z*, 705 *m/z* and 615 *m/z* gave the best linearity and were subsequently used for quantification analysis.

### Determination of microbial growth and surface tension

Microbial growth was determined by measuring the optical density of the culture medium (OD_600_) at regular intervals during the fermentation using a Novaspec II spectrophotometer (Pharmacia Biotech). Media with OD_600_ values higher than 1 were diluted, and the results obtained were multiplied by the dilution factor. Values obtained were used to plot growth curves to determine the differences in growth patterns of the strains used. For comparison, viable counts of the triple mutant and wild-type strains were performed at various times during the fermentation using the Miles and Misra technique (Miles et al. 1938).

Surface tension measurements were also carried out at intervals during the fermentation as a measure of biosurfactant production. Measurements were carried out on a KRUSS KI0ST tensiometer with a platinum ring using 10-ml supernatants from the fermentation broth.

### Measurement of glycerol concentration

Glycerol concentration was measured during fermentation using a spectrophotometric method for the determination of free glycerol concentration (Bondioli and Della Bella 2005). This method involves the formation of formaldehyde from glycerol through a series of reaction involving periodate oxidation. The formaldehyde produced is then converted to 3,5-diacetyl-1,4-dihydrolutidine via Hantzsch’s reaction in the presence of acetylacetone and ammonium acetate (Bondioli and Della Bella 2005).

Cell-free supernatant samples were diluted in a working solvent which consisted of 1:1 *v/v* distilled water and 95% ethanol. Two millilitres of diluted sample was then transferred to a 10-ml tube followed by the addition of 1.2 ml of 10 mM sodium periodate solution. The solution was manually mixed by shaking for 30 s before adding 1.2 ml of a 0.2-M acetylacetone solution. This was then transferred to a water bath maintained at 70 °C for 1 min, stirring manually. Samples were immediately cooled by immersing tubes in a beaker of tap water after the reaction time. The optical density of the samples was determined at 410 nm using a Novaspec II spectrophotometer (Pharmacia Biotech), using a blank prepared by using 2 ml of working solvent. A standard curve was also prepared using different concentrations of glycerol (BDH Chemicals).

Both sodium periodate and acetylacetone solutions were prepared daily and used immediately after preparation. Sodium periodate solution was prepared by dissolving 21 mg of sodium meta-periodate (Sigma, UK) in 5 ml of 1.6 M acetic acid, followed by the addition of 5 ml of 4.0 M ammonium acetate (Sigma, UK), while acetylacetone solution was prepared by first mixing 5 ml of 1.6 M acetic acid with 5 ml of 4.0 M ammonium acetate, followed by the addition of 200 μl acetylacetone.

### Polyhydroxyalkanoate extraction

Polyhydroxyalkanoate (PHA) extraction was carried out as described previously (Tripathi et al. 2012) with slight modification, from biomass obtained by centrifugation of a 30-ml fermentation broth at 13,000 *g* for 15 min. Biomasses were frozen at − 20 °C and then lyophilised for 48 h. The cell dry weight of the biomass was recorded; PHA was extracted from lyophilised cells by adding 10 ml of chloroform to cells at 95 °C for 4 h. Cells were filtered off, and the PHA was extracted using a 10-fold volume of ice-cold methanol. The precipitated PHAs were collected by centrifugation and dried under a stream of nitrogen gas to constant weight.

### PHA characterisation

#### Nuclear magnetic resonance

Extracted polymers were identified by nuclear magnetic resonance (NMR) analysis. The NMR spectra were recorded using a Bruker Avance II 600 MHz with Ultrashield Plus Magnets. The ^1^H and ^13^C NMR spectra were run in CDCl_3_ using tetramethylsilane (TMS) as an internal standard.

#### Differential scanning calorimetry

Differential scanning calorimetry (DSC) measurements were taken with a TA DSC 2010 apparatus (TA Instruments, New Castle, DE) with a temperature range of − 50 to + 220 °C. The glass transition temperatures (*T*_g_) were determined at a heating rate of 20 °C/min. The instrument was calibrated with high-purity indium and gallium. The crystallinity of the polymers was calculated using Eq. 1, where 퐶 (%) is the percentage crystallinity, ${\Delta  H}_{\mathrm{m}}^{\mathrm{m}}$ is the measured melting enthalpy (J/g) and $\Delta  {H}_{\mathrm{m}}^{100\%}$ is the melting enthalpy for fully crystalline polyhydroxybutyrate (PHB) (146 J/g) (Radecka et al. 2016).

1 $$ C\left(\%\right)=\frac{{\Delta  H}_{\mathrm{m}}^{\mathrm{m}}}{\Delta  {H}_{\mathrm{m}}^{100\%}} $$

### Determination of pathogenicity

Pathogenicity assays were carried out on both *B. thailandensis* E264 wild-type and triple mutant strains. Analyses were carried out using the *Galleria mellonella* model as previously described (Irorere et al. 2018). *G. mellonella* were purchased from Pets at Home (Coleraine, Northern Ireland, UK), and 10 larvae were used per strain, in triplicate. Each larva was injected with 100 cfu of each strain in 20 μl of phosphate-buffered saline (PBS), with sterile PBS used as a negative control. Larvae were incubated for 48 h at 37 °C in perforated petri dishes.

### Statistical analysis

GraphPad Prism version 6 was used to analyse results of this study. Unless stated otherwise, analyses were carried out using one- or two-way ANOVA with Tukey’s multiple comparison (*P* ≤ 0.05).

## Results

### Effect of QS mutations on cell growth

To determine the effect of QS mutations on *B. thailandensis*, strains were grown in nutrient broth supplemented with 4% glycerol at 30 °C and 200 rpm for 240 h. During this period, growth was assessed by measuring the absorbance at OD_600_. The initial average cell concentration at the start of fermentation was approximately OD_600_ of 0.23. Single mutant strains (S*ΔbtaI1*, S*ΔbtaI2* and S*ΔbtaI3*) grew to an OD_600_ of 8.89, while double mutant strains (D*ΔbtaI2ΔbtaI3*) grew to an OD_600_ of 9.22 with a maximum OD_600_ of 5.08 recorded for the triple mutant strain (T*ΔbtaI1ΔbtaI2ΔbtaI3*) (Fig. S2a). Tukey multiple comparison in two-way ANOVA showed no significant difference in the growth of all single mutant strains compared with the wild type throughout the fermentation period. However, the cell concentration of the double mutant strain was significantly higher (*P* < 0.05) from 72 h compared with the wild type, while the cell concentration of the triple mutant was significantly lower (*P* < 0.05) from 48 h of fermentation compared with both the wild-type and the single mutant strains. Additionally, the triple mutant strain reached stationary phase at 24 h of growth, while the wild-type and other mutants reached stationary phase between 72 h and 96 h of growth (Fig. S2a).

The triple mutant strain was subsequently selected for complementation analysis, as it showed the most significant difference in cell growth when compared with the wild type. The initial complementation study was carried out in fresh nutrient broth supplemented with 40% filter-sterilised spent media from an overnight culture of wild-type *B. thailandensis* and 4% glycerol. Results showed that the triple mutant strain had similar growth patterns and microbial concentration throughout the fermentation, compared with the wild-type strain used as a control (Fig. S2b). The cell concentration of the triple mutant reached OD_600_ of 7.70, similar to levels observed in wild-type cultures with or without spent medium supplementation (OD_600_ 8.22 and 7.02 respectively) (Fig. S2a-b).

**Fig. 1:**
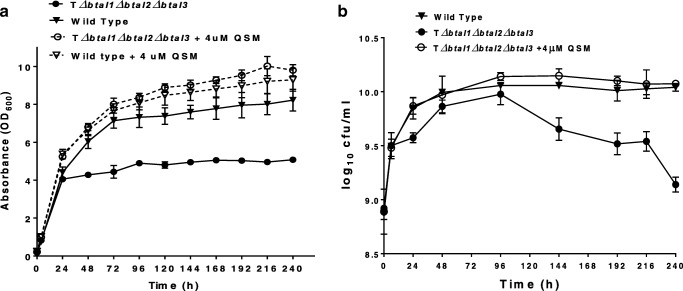
Quorum sensing triple mutant experience reduced growth and death phases compared with the wild type. Growth of wild-type and triple mutant strains measured by either optical density (**a**) or viable cell count (**b**) in non-supplemented nutrient media or nutrient media supplemented with 4 μM each of 3OHC_10_HSL, 3OHC_8_HSL and C_8_HSL. Viable cell numbers in cfu/ml were converted to log_10_ values, which is represented in the *Y*-axis as log_10_ cfu/ml. All experiments were carried out in triplicate (*n* = 3)

Based on the above result, a further complementation study was designed using nutrient broth supplemented with 4 μM each of the three QS signal molecules of *B. thailandensis* E264 (3OHC_10_-HSL, 3OHC_8_-HSL and C_8_-HSL). Cell growth of both the wild-type and the triple mutants in supplemented media was analysed, and results were compared with those obtained in non-supplemented media (Fig. 1a). Similar to results observed with the initial complementation study, the growth curve of the triple mutant strain was restored to patterns similar to that of the wild type after medium supplementation with synthetic QS signalling molecules. Furthermore, the cell concentration of the triple mutant (OD_600_ of 9.80) after 240 h of fermentation was at levels comparable to media supplemented (OD_600_ of 9.22) and non-supplemented (OD_600_ of 8.22) wild-type cultures (Fig. 1a).

Due to the significantly lower OD_600_ recorded for the triple mutant strain compared with the wild-type and other mutants, viable cell count was carried out for both the triple mutant strain, wild type strain and triple mutant strain in media complemented with synthetic QS signal molecules. In non-complemented media of the triple mutant strain, the maximum number of viable cells recorded was log_10_ 9.97 cfu/l at 96 h of fermentation. After this, the cells went into death phase with a final viable cell number of 9.14 cfu/ml recorded at the end of the fermentation (240 h). Neither the wild-type nor triple mutant strain in complemented media went into death phase; they both maintained the stationary phase from 48 h and had viable cell numbers of log_10_ 10.04 CFU/ml and log_10_ 10.07 cfu/ml respectively at the end of fermentation (Fig. 1b). Additionally, the viable count at 24 h in the triple mutant strain was observed to be significantly lower than the wild-type strain, although the difference at 48 h and 96 h were not significant compared with either the wild-type or medium-complemented triple mutants (Fig. 1b).

### Glycerol consumption

**Fig. 2:**
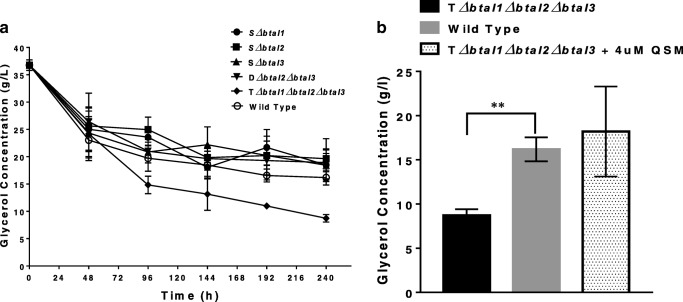
There is increased glycerol metabolism by quorum sensing triple mutant strain compared with wild type or other quorum sensing mutants. **a** Glycerol concentration of wild-type *B. thailandensis* and all quorum sensing mutants in non-supplemented nutrient broth. **b** Glycerol concentration of triple mutant strain in non-supplemented nutrient broth and nutrient media supplemented with 4 μM each of 3OHC_10_HSL, 3OHC_8_HSL and C_8_HSL, in comparison to wild type. Glycerol concentrations in media supernatants were assessed using a spectrophotometric method described previously (Bondioli and Della Bella 2005), and all experiments were conducted in triplicate (*n* = 3). Differences in glycerol concentration between the wild type and the triple mutant were analysed using unpaired *t* test in GraphPad Prism 7. WT = wild type and TM = triple mutant

To see if the growth limitation observed in the triple mutant strain is due to their inability to utilize available nutrient, we measured the change in glycerol concentration throughout the fermentation period in all mutants’ strains and the wild type. Surprisingly, we observed that glycerol concentration was consistently lower in the triple mutant compared with the WT or other mutant strains used in this study during the stationary growth phase (Fig. 2a). However, the glycerol concentrations of all the single and double mutant strains were not significantly (*P* ≥ 0.05) different from the wild type at 240 h of fermentation (Fig. 2a). The mean glycerol concentration of the triple mutant at 240 h was 8.76 g/l, which was significantly lower (*P* < 0.05) than the concentration of the wild type at 16.19 g/l and other mutants at concentrations between 18.45 and 19.64 g/l (Fig. 2a). This trend was observed from 96 h of growth, with the triple mutant consistently showing lower concentration of glycerol compared with either the wild-type or other mutant strains. When the triple mutant strains were grown in media supplemented with QS signal molecules, the glycerol consumption was observed to be similar to that of the wild type with a final concentration of 18.2 g/l at 240 h (Fig. 2b).

### Surface tension analysis and rhamnolipid production

**Fig. 3:**
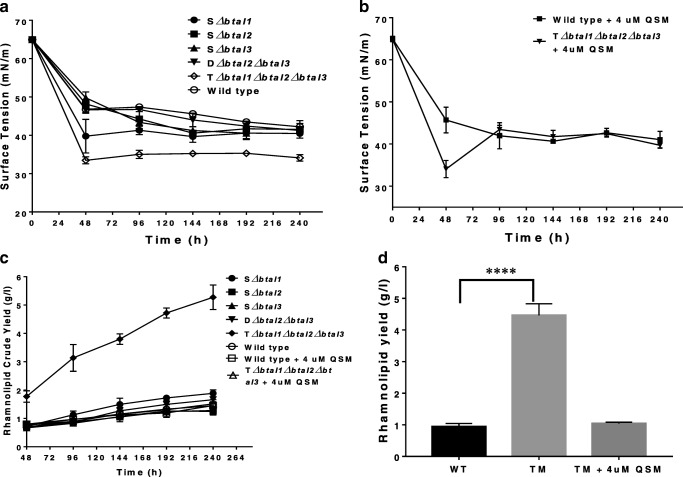
Rhamnolipid production by quorum sensing triple mutant strain of *B. thailandensis* is significantly higher compared with the wild type or other mutant strains. **a** Surface tension of the different quorum sensing mutants of *B. thailandensis* and the wild type, showing lower surface tension of the triple mutant media compared with other strains. **b** Restoration of surface tension of *B. thailandensis* triple mutant media to levels comparable to the wild type when nutrient broth is supplemented with 4 μM each of 3OHC_10_HSL, 3OHC_8_HSL and C_8_HSL. **c** Crude rhamnolipid yield of all quorum sensing mutants of *B. thailandensis* and the wild type, showing a significantly higher yield of the triple mutant compared with other strains including the wild type. **d** Rhamnolipid yield of quorum sensing triple mutant grown in non-supplemented and synthetic HSLs supplemented nutrient broth at 240 h compared with wild type. All experiments were carried out in triplicates (*n* = 3), and the differences in rhamnolipid yield between the wild type and the triple mutant were analysed using the unpaired *t* test in GraphPad Prism 7

As an indication of biosurfactant production, the media supernatants were collected at time intervals to observe reduction in surface tension. All single and the double mutant strains were able to reduce the surface tension of the production medium from 65.0 mN/m to approximately 41.0 mN/m at 240 h of fermentation, which is similar to that of the wild type (Fig. 3a). However, rather surprisingly, the triple mutant strain reduced the surface tension of the fermentation medium from 65 to 34.1 mN/m at 240 h of fermentation (Fig. 3a).

Complementation analysis in nutrient broth supplemented with 40% spent media was observed to restore the surface tension of the triple mutant to levels similar to those of the wild type grown in both 40% spent media supplemented and non-supplemented nutrient broth (Fig. S3a). This restoration was also observed in nutrient broth supplemented with 4 μM each of the three QS signal molecules (Fig. 3b). However, in the latter, the surface tension at 48 h was lower than that of the wild type (supplemented and non-supplemented) and comparable to the triple mutant strain grown in non-supplemented media (Fig. 3b).

Following surface tension analysis, rhamnolipid extraction was carried out at different stages of fermentation and yields were compared with those obtained from the wild type (Fig. 3c). At 48 h of fermentation, rhamnolipid crude yields in the three single (S*ΔbtaI1*, S*ΔbtaI2* and S*ΔbtaI3*) and double (D*ΔbtaI2ΔbtaI3*) mutant strains at 0.72 g/l, 0.70 g/l, 0.67 g/l and 0.77 g/l respectively were comparable to that of the wild type at 0.79 g/l. This trend continued throughout the growth cycle (96 h, 144 h, 192 h and 240 h), with no significant (*P* ≥ 0.05) differences observed in the crude yield of the single and double mutants compared with the wild type (Fig. 3c). Unexpectedly, we observed that the rhamnolipid crude yield from the triple mutant (T*ΔbtaI1ΔbtaI2ΔbtaI3*) was significantly higher (*P* < 0.05) than that of the wild type and all other mutant strains. At 240 h of fermentation, the rhamnolipid crude yield of the triple mutant was 5.27 ± 0.25 g/l, while that of the wild type at the same time was 1.45 ± 0.12 g/l (Fig. 3c). Complementation of the fermentation broth with 40% spent culture from the wild-type strain led to a reduction in the rhamnolipid crude yield of the triple mutant to 1.16 ± 0.08 g/l at 240 h of fermentation (Fig. S2b). Similarly, complementation with 4 μM each of the three different QS signal molecules, resulted in a reduction of rhamnolipid crude yield to comparable levels as the wild type (with or without QS signal molecule complementation) throughout the fermentation, with a final crude yield of 1.51 ± 0.05 g/l at 240 h (Fig. 3c).

To be sure that the high rhamnolipid yield observed in gravimetric analysis of rhamnolipid crude yield is not due to high fatty acid content in the extract, further characterisation of rhamnolipid yield at 240 h was carried out by LC/MS. Results showed yields of 0.94 ± 0.06 g/l, 1.04 ± 0.03 g/l and 4.46 ± 0.345 g/l for the wild type, medium-complemented triple mutant and the triple mutant in non-complemented media respectively (Fig. 3d).

To see if the increase in rhamnolipid production had any effect on the pathogenicity of *B. thailandensis*, the *Galleria mellonella* model of pathogenicity assessment was used to compare the pathogenicity of wild type and the triple mutant strain. Larvae were infected with 100 cfu each of either wild-type or triple mutant strain. The result presented showed that both strains had up to 94% survival of the larvae at 18 h of infection. However, the triple mutant strains had about 33% survival at 30 h, lower than larvae infected with the wild-type strain, with 90% survival. However, there was a 100% mortality or 0% survival after 48 h of infection with either strain (Fig. S3c).

### Rhamnolipid characterisation

The purified rhamnolipid from the wild type and the triple mutant strain were analysed by LC/MS, and the relative proportions of the various congeners in both strains were compared. Characterisation was carried out on purified rhamnolipid from independent triplicate experiment, and results are summarised in Table 2, with similar congeners observed in both strains. As expected, the dirhamnolipids were the most abundant in the wild-type strain compared with the monorhamnolipids (67.5 ± 5.3 to 32.5 ± 3.1, respectively), with a lipid chain length of C_14_–C_14_ found to be the most abundant mono- and dirhamnolipid congeners. In contrast, we observed that monorhamnolipids were the most abundant congeners compared with dirhamnolipids (53.7 ± 5.7 to 46.3 ± 6.3 respectively) in the triple mutant strain. However, rhamnolipids with a lipid chain length of C_14_–C_14_ remained the most abundant mono- and dirhamnolipids congeners, as observed in the wild-type strain.

When we supplemented the rhamnolipid production medium with QS signal molecules, results indicate a similar trend of rhamnolipids compared with triple mutants grown in supplemented media from the wild type; dirhamnolipids were observed as the most abundant congeners compared with monorhamnolipids. Additionally, the relative abundance of dirhamnolipid in the triple mutant grown in QS signal molecule supplemented media was observed to be significantly higher than that of the wild type grown in non-supplemented media (86.8 ± 7.6 and 67.5 ± 5.3 respectively). However, it was similar to the wild type grown in QS signal molecule–supplemented media (90.5 ± 20.2) (Table 2).

### PHA production and characterisation

**Fig. 4:**
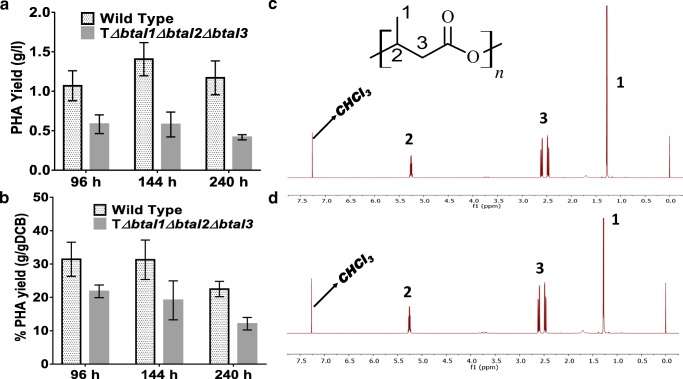
Polyhydroxyalkanoate (PHA) yield is lower in *B. thailandensis* quorum sensing triple mutant compared with wild type. **a**, **b** PHA yield (g/l) and percentage PHA productivity (g/gDCB) respectively at different stages of fermentation of wild-type and triple mutant strains of *B. thailandensis*. **c**, **d** H′ NMR of polyhydroxybutyrate (PHB) produced by wild-type and triple mutant strains of *B. thailandensis* E264 respectively. PHA was extracted from lyophilised biomass by solvent extraction with chloroform followed by precipitation with ice-cold methanol. Results presented are biological triplicates (*n* = 3)

To investigate if increased rhamnolipid production is due to reduction in the storage of carbon as polyhydroxyalkanoate (PHA), the PHA yield by the QS complete mutant was assessed and compared with that of the wild type. PHA yield was observed to peak at 144 h of fermentation in both the wild type and mutant strains, with the wild-type strain consistently having a significantly higher (*P* < 0.05) PHA yield compared with the mutant strain (Fig. 4a). The highest yields observed were 1.41 ± 0.12 g/l and 0.58 ± 0.09 g/l, from the wild type and the triple mutant strains respectively. To identify if this significantly lower yield can be accounted for by the lower growth rate observed in the triple mutant strain, the percentage yield per cell dry weight was calculated. The wild-type strain also had a consistently higher percentage yield (g/gCDW) compared with the triple mutant strain, with the highest recorded percentage yield of 31.4% and 21.8% respectively at 96 h of fermentation (Fig. 4b).

Both ^1^H and ^13^C NMR confirmed that poly-3-hydroxybutyrate was the primary PHA produced by both the complete mutant and the wild-type strain of *B. thailandensis* (Fig. 4c, d and Fig. S4a-b). DSC traces of PHA from wild-type and complete mutant strains showed that the glass transition temperatures were 3.2 °C and 1.2 °C respectively, with melting temperatures of 172.1 °C and 165.3 °C respectively. Additionally, the crystallinity of the polymers were 52.2% and 51.2% for wild-type and complete mutant strains respectively.

## Discussion

The classification of *B. thailandensis* E264 as a biosafety level I or a putative non-pathogen makes it an attractive substitute for rhamnolipid production, compared with *P. aeruginosa* which is an opportunistic pathogen or biosafety level II organism (Brett et al. 1998; Irorere et al. 2017). However, low levels of rhamnolipid production and the significantly longer fermentation time required to produce substantial quantities of rhamnolipid are major factors affecting the implementation of *B. thailandensis* in the industrial production of rhamnolipid. Iron limitation in the production media and the introduction of exogenous quorum sensing molecules have been suggested as ways of inducing early rhamnolipid synthesis and improving overall rhamnolipid yield (Irorere et al. 2018). This study was designed to assess the effect of quorum sensing mutations on rhamnolipid production by *B. thailandensis* E264 and subsequently to determine the effect, if any, of exogenous addition of quorum sensing molecules on rhamnolipid production by *B. thailandensis*.

*B. thailandensis* is known to have three different quorum sensing circuits, BtaI1–BtaR1, BtaI2–BtaR2 and BtaI3-BtaR3, that respond to or direct the synthesis of C_8_-HSLs, 3OHC_10_-HSLs and 3OHC_8_-HSLs (Chandler et al. 2009; Le Guillouzer et al. 2017), with two orphan LuxR signal receptors, lacking the known LuxI signal synthase, BtaR4 and BtaR5. Mutations in any or all of the *LuxI* signal synthase will result in the cell’s inability to synthesize the corresponding signal molecules. Subsequently, the corresponding LuxR signal receptors are orphaned and unable to direct the synthesis of the particular signal molecule, unless there is the addition of respective exogenous signal molecules. Based on this, we assessed the rhamnolipid production of either single (S*ΔbtaI1*, S*ΔbtaI2* and S*ΔbtaI3*), double (D*ΔbtaI2ΔbtaI3*) or triple (T*ΔbtaI1ΔbtaI2ΔbtaI3*) mutant strains of *B. thailandensis* E264 to see the effect of quenching single or multiple QS circuits on rhamnolipid synthesis in *B. thailandensis* E264*.*

While assessing the growth of *B. thailandensis* during rhamnolipid production, we observed that the triple mutant strain reached stationary phase earlier and at significantly lower cell density compared with both the wild type and the double or all three single mutant strains (Fig. S1a and Fig. 1a). This was a surprising and unexpected discovery as QS mutations or quorum quenching (QQ) in most microbial strains including *Vibrio harveyi*, *Yersinia ruckeri*, *Sodalis praecaptivus*, *P. aeruginosa* and *Burkholderia glumae* have been shown to either have no effect or to enhance microbial growth or growth rate under different experimental conditions (Enomoto et al. 2017; Ishida et al. 2007; Nackerdien et al. 2008; Nickzad and Déziel 2016; Torabi Delshad et al. 2018). In the above studies, QS mutants or WT strains growing in the presence of quorum quenching (QQ) molecules showed comparable or enhanced growth compared with the wild type.

One report that showed growth limitation as a consequence of QS mutation was Goo et al. (2012). In this report, mutant strains of *B. glumae, B. pseudomallei* and *B. thailandensis* deficient in C_8_-HSL synthesis experienced a rapid decline in cell population, due to increased alkalinity of the media, as the cells enter into late stationary phase in a nutrient-rich medium, compared with the WT (Goo et al. 2012). However, a more recent study did not observe any effect on growth of a C_8_-HSL deficient mutant of *B. glumae* (*tofl*^*−*^) in both minimal and rich media (Nickzad and Déziel 2016). Similarly, no decline in the cell population of a C_8_-HSL-deficient mutant of *B. thailandensis* (S*ΔbtaI1*) was observed in our study, with a sustained stationary phase observed throughout the period of fermentation (Fig. S1a).

It has previously been reported that quorum sensing mutations affect self-aggregation of *B. thailandensis* in minimal media (Chandler et al. 2009). To ensure that the lower OD_600_ observed in the triple mutant is not a result of differences in self-aggregation in the cultures, viable cell count was carried out. Results showed that cell count of the triple mutant was significantly lower at 24 h. However, the differences in cell count compared with the wild type and the triple mutant growing in the presence of HSLs were not significant at 48 h and 96 h, with the triple mutant strain going into death phase after 96 h (Fig. 1b). The fact that the death phase observed during viable cell count is not seen in the OD reading suggests the potential of increased accumulation of cell exudates in the media, including rhamnolipid; this may also explain the decline in cell number due to cell toxicity.

However, it is also possible that the decline in cell number is due to the inability of the triple mutant to utilise available substrates as previously reported in other microbial strains including *P. aeruginosa* (Antunes et al. 2007; Pearson et al. 1997; Studer et al. 2008). To see if this is true for *B. thailandensis*, we studied the change in glycerol concentration throughout the fermentation period. Surprisingly, we observed that glycerol concentration was consistently lower in the triple mutant compared with the wild-type or other mutant strains used in this study during stationary growth phase (Fig. 2a). Both growth and glycerol utilisation were restored to comparable levels as the wild type when triple mutant strains are grown in media supplemented with wild-type spent media or 4 μM of each of the 3 QS signal molecules. This implies that glycerol was utilised much faster in the triple mutant compared with both the wild-type and other mutant strains. This result is in agreement with a previous study in which QS was shown to have no effect on the metabolic diversity of *B. thailandensis* as it does in *P. aeruginosa* (Chandler et al. 2009). Indeed, QS was reported to negatively influence the rate of respiration of *B. thailandensis* on most carbon, nitrogen and phosphorus sources (Chandler et al. 2009). Collectively, these results suggest that growth limitation observed in QS triple mutant is not due to their inability to utilize available carbon or energy sources.

As QS is known to control the gene expression of a wide range of secondary metabolites in *B. thailandensis* (Majerczyk et al. 2014), one possible explanation for growth limitation could be the overexpression and/or continuous activation of ‘unnecessary’ genes, thus resulting in increased demand on the cell’s energy supply, thus trading off growth for continuous production of metabolites. This may also explain the increased glycerol metabolism by the cell. As to why this effect is not observed in single or double mutant strains, reports have shown an overlap in genes regulated by the different QS circuits (Majerczyk et al. 2014). Thus, some of the effect of mutations in one QS circuit can be compensated for by another.

Rhamnolipid extraction and quantification showed that the triple mutant had a significantly higher crude rhamnolipid yield compared with both the wild type and other mutant strains, while the differences in crude yield of the single and double mutants were not significant compared with the wild type (Fig. 3c). Thus, only the wild type and the triple mutant strain were subsequently selected for rhamnolipid quantification by HPLC-MS. Data obtained from LC-MS showed that the QS triple mutant produced over 4 times more rhamnolipid compared with the wild type, with complementation restoring rhamnolipid production to similar levels as the wild type (Fig. 3d). This therefore confirms that rhamnolipid production is significantly higher in triple mutant compared with the wild type.

We also wanted to know if the increase in glycerol consumption led to the accumulation of more energy storage by the cell in the form of PHA. To do this, PHAs were extracted and quantified from both the wild-type and triple mutant strains. This was also carried out because previous studies on *P. aeruginosa* have shown that QS mutants have significantly lower levels of PHA production (Xu et al. 2011). We have also previously shown that PHA mutant strains of *B. thailandensis* produce significantly higher quantities of rhamnolipid compared with WT (Funston et al. 2017). Hence, it is possible that carbon is pulled away from energy storage towards rhamnolipid biosynthesis. We therefore hypothesise that the increased rhamnolipid production is in part due to a reduction in PHA biosynthesis, increased glycerol metabolism and reduced growth.

As expected, PHA production was consistently higher in the WT compared with the triple mutant at different stages of extraction (Fig. 4a). To be sure that this higher PHA yield is not due to reduction in cell biomass, we calculated the percentage PHA per cell (g/gCDB). Results showed that the percentage PHA (g/gDCB) of the triple mutant was also consistently lower than the WT (Fig. 4b). QS does not, however, have any effect on the structural properties of PHA produced by *B. thailandensis* in this study, with PHB identified as the main product in both strains (Fig. 4c, d). However, DSC characterisation showed reductions in the glass transition and melting temperatures of PHA from triple mutant compared with the wild-type strain. The thermal properties of PHA from both strains were however consistent with those of PHB reported previously (Irorere et al. 2014; Radecka et al. 2016).

As rhamnolipid is a known virulence factor in many Gram-negative bacterial strains, including *P. aeruginosa*, *B. glumae* and *B. pseudomallei* (Häuβler et al. 2003; Nickzad et al. 2015; Zulianello et al. 2006), we hypothesise that the increased rhamnolipid yield in the triple mutant strain might also increase their virulence. Using the *G. mellonella* model of pathogenicity, we observed a similar pattern in the mortality of *G. mellonella* by both triple mutant and WT strains. However, we observed a higher mortality at 30 h of larval inoculation after infection with the triple mutant compared with the WT. However, this is not comparable to the pathogenicity of *P. aeruginosa* which showed a 100% mortality of *G. mellonella* larvae at 18 h of inoculation under similar experimental conditions (Irorere et al. 2018).

Indeed, an earlier study has shown that QS is not a virulence determinant in *B. thailandensis*, as it is in the closely related *B. pseudomallei*, using the mouse model (Chandler et al. 2009). In their study, Chandler et al. did not observe any significant difference in the pathogenicity of triple mutant *B. thailandensis* strain on mice compared with the WT (Chandler et al. 2009). Together, these results suggest that QS has a negligible effect on *B. thailandensis* virulence. This is contrary to results reported in other bacterial strains including the closely related *B. pseudomallei*, where QS mutations are known to attenuate virulence (Ulrich et al. 2004). Hence, the negligible increase in pathogenicity observed in *B. thailandensis* further supports the fact that QS is a negative regulator of rhamnolipid production in *B. thailandensis* compared with other microbial strains discussed earlier.

Purified rhamnolipids from both triple mutant and WT were characterised by LC/MS. As expected, dirhamnolipids were the most predominant congeners in WT *B. thailandensis*. However, monorhamnolipids were observed as the most predominant congeners in the triple mutant strain (Table 1). These results are an average of rhamnolipid from biological triplicates to ensure that relative abundances are not due to experimental errors. Complementation of the media with synthetic HSLs in the triple mutant strain led to the restoration of dirhamnolipids as the predominant rhamnolipid congeners (Table 2), thus confirming that QS does indeed affect rhamnolipid congener distribution in *B. thailandensis*, favouring the production of dirhamnolipids. The increased abundance of monorhamnolipids in the triple mutant compared with the wild type may account for the lower surface tension observed in the former compared with the latter (Fig. 3a). This is because monorhamnolipids are known to reduce surface tension to values lower than dirhamnolipids (Dubeau et al. 2009; Tiso et al. 2017).

**Table 1:** Average relative abundance of purified rhamnolipid congeners produced by wild-type and triple mutant of *B. thailandensis* E264. Results presented are averages of analysis from biological triplicates of rhamnolipid extracts, and the error values are indicative of the standard deviation of the mean. ND = below the level of detection

Rhamnolipid congener	Pseudomolecular ion (*m*/*z*)	Retention time (min)	Relative abundance (%)	
			Wild type	Triple mutant
Rha–C_10_–C_12_/C_12_–C_10_	531	23.17	ND	1.31 ± 0.14
Rha–C_12_–C_12_/C_10_–C_14_/C_14_–C_10_	559	25.58	1.65 ± 0.27	6.42 ± 0.78
Rha–C_12_–C_14_/C_14_–C_12_	587	27.72	5.35 ± 0.49	18.01 ± 1.86
Rha–C_14_–C_14_	615	29.32	23.65 ± 1.62	26.88 ± 2.84
Rha–C_14_–C_16_/C_16_–C_14_	643	30.73	1.85 ± 0.67	1.04 ± 0.11
Total monorhamnolipid			32.50 ± 3.05	53.66 ± 5.73
Rha–Rha–C_10_–C_12_/C_12_–C_10_	677	19.16	1.35 ± 0.37	2.2 ± 0.43
Rha–Rha–C_12_–C_12_/C_10_–C_14_/C_14_–C_10_	705	22.81	8.69 ± 1.56	11.17 ± 2.59
Rha–Rha–C_12_–C_14_/C_14_–C_12_	733	25.19	15.32 ± 0.96	15.62 ± 2.14
Rha–Rha–C_14_–C_14_	761	27.21	36.99 ± 1.91	16.03 ± 0.94
Rha–Rha–C_14_–C_16_/C_16_–C_14_	789	28.94	5.16 ± 0.48	1.32 ± 0.23
Total dirhamnolipid			67.51 ± 5.28	46.34 ± 6.33

**Table 2:** Average relative abundance of purified rhamnolipid congeners produced by wild-type and triple mutants of *B. thailandensis* E264 grown in the presence of 4 μM each 3OHC_10_HSL, 3OHC_8_HSL and C_8_HSL. Results presented are averages of analysis from biological triplicates of rhamnolipid extracts, and the error values are indicative of the standard deviation of the mean. ND = below the level of detection

Rhamnolipid congener	Pseudomolecular ion (m/z)	Retention time (min)	Relative abundance (%)	
			Wild type	Triple mutant
Rha–C_12_–C_14_/C_14_–C_12_	587	27.72	1.49 ± 0.50	1.77 ± 0.51
Rha–C_14_–C_14_	615	29.32	7.49 ± 4.1	10.97 ± 2.68
Rha–C_14_–C_16_/C_16_–C_14_	643	30.73	0.52 ± 0.23	0.51 ± 0.17
Total monorhamnolipid			9.50 ± 4.85	13.25 ± 3.36
Rha–Rha–C_10_–C_12_/C_12_–C_10_	677	19.16	1.91 ± 0.76	2.16 ± 0.47
Rha–Rha–C_12_–C_12_/C_10_–C_14_/C_14_–C_10_	705	22.81	12.72 ± 6.74	11.31 ± 3.76
Rha–Rha–C_12_–C_14_/C_14_–C_12_	733	25.19	22.29 ± 4.59	17.39 ± 1.23
Rha–Rha–C_14_–C_14_	761	27.21	49.68 ± 6.18	50.61 ± 1.15
Rha–Rha–C_14_–C_16_/C_16_–C_14_	789	28.94	3.90 ± 1.92	5.3 ± 1.03
Total dirhamnolipid			90.5 ± 20.19	86.77 ± 7.64

Additionally, we observed a significantly higher dirhamnolipid proportion in the wild-type strain grown in the presence of exogenous HSLs compared with the wild-type strain grown without exogenous HSLs. To the best of our knowledge, this is the first report implicating rhamnolipid congener distribution with QS. However, no significant difference was observed in the relative abundance of dirhamnolipid in wild-type and triple mutant strains grown in the presence of exogenous HSLs.

In conclusion, our report has shown that triple QS mutation in *B. thailandensis* produced higher rhamnolipid yield compared with the wild type, partly by increased metabolism and reduced accumulation of storage material in the form of PHA. Additionally, QS was also shown to affect rhamnolipid congener distribution by favouring the synthesis of dirhamnolipids over monorhamnolipids. Furthermore, introduction of acyl-HSLs in production media increased the relative abundance of dirhamnolipids compared with monorhamnolipids. Thus, QS and acy-HSLs could be a way to direct the synthesis of desired rhamnolipid congeners in *B. thailandensis*.

## Electronic supplementary material

ESM 1 (PDF 614 kb)
