# Supplementary material for: Quorum sensing as a potential target for increased production of rhamnolipid biosurfactant in Burkholderia thailandensis E264
Source: Appl Microbiol Biotechnol. 2019 Jun 21;103(16):6505–17. doi: 10.1007/s00253-019-09942-5 (PMC6667413; doi:10.1007/s00253-019-09942-5)
Supplement: Supplementary file 1 — (PDF 614 kb) [file 253_2019_9942_MOESM1_ESM.pdf]

APPLIED MICROBIOLOGY AND BIOTECHNOLOGY

SUPPLEMENTARY MATERIALS FOR

Quorum sensing as a potential target for increased production of rhamnolipid biosurfactant in *Burkholderia thailandensis* E264.

Irorere U. Victor [1] (Irorere-V@ulster.ac.uk)

Michal Kwiencien [2] (kwiecienmichal@poczta.fm)

Lakshmi Tripathi [1] (L.tripathi@ulster.ac.uk)

Diego Cobice [1] (D.cobice@ulster.ac.uk)

Stephen McClean [1] (S.mcclean@ulster.ac.uk)

Roger Marchant [1] (R.marchant@ulster.ac.uk)

Ibrahim M. Banat [1*] (im.banat@ulster.ac.uk)

[1] School of Biomedical Sciences, Faculty of Life and Health Sciences, Ulster University, Coleraine, BT52 1SA, Northern Ireland, UK.

[2] Centre of Polymer and Carbon Materials, Polish Academy of Sciences, 34, M. Curie-Skłodowska St., 41-819 Zabrze, Poland.

*Correspondence to: Tel: +44 28 7012 3062; Email: im.banat@ulster.ac.uk (Prof Ibrahim Banat)

Table S1: Microbial strains and genotypic descriptions used in this study

**Bacteria strain**	**Genotypic description**	**Designated name**	**Reference**
E264	Wild type	Wild type	Brett et al., 1998
JBT101	*ΔbtaI1*	Single mutant (S*ΔbtaI1*)	Chandler et al., 2009
JBT102	*ΔbtaI2*	Single mutant (S*ΔbtaI2*)	Chandler et al., 2009
JBT103	*ΔbtaI3*	Single mutant (S*ΔbtaI3*)	Chandler et al., 2009
JBT104	*ΔbtaI2ΔbtaI3*	Double mutant (D*Δbta2ΔbtaI3*)	Chandler et al., 2009
JBT112	*ΔbtaI1ΔbtaI2ΔbtaI3*	Triple mutant T*ΔbtaI1ΔbtaI2ΔbtaI3*	Chandler et al., 2009

Table S2: Gradient profile used for HPLC analysis. A and B represent water and acetonitrile respectively, used as mobile phases.

**Time**	**A (%)**	**(B %)**
0.00	80	20
4.00	80	20
30.00	0	100
35.00	0	100
35.10	80	20
20.10	80	20

Figure S1: Linear regression of rhamnolipid concentration and the area of the ratios of sum of the selected pseudomoleular ions and the internal standard. Different pseudomolecular ions representing monorhamnolipid and dirhamnolipid were initially used as quantifier ions, to study their linearity with increasing rhamnolipid concentrations. The pseudomolecular ions 733 *m/z*, 705 *m/z* and 615 *m/z* gave the best linearity and were subsequently used for quantification analysis.

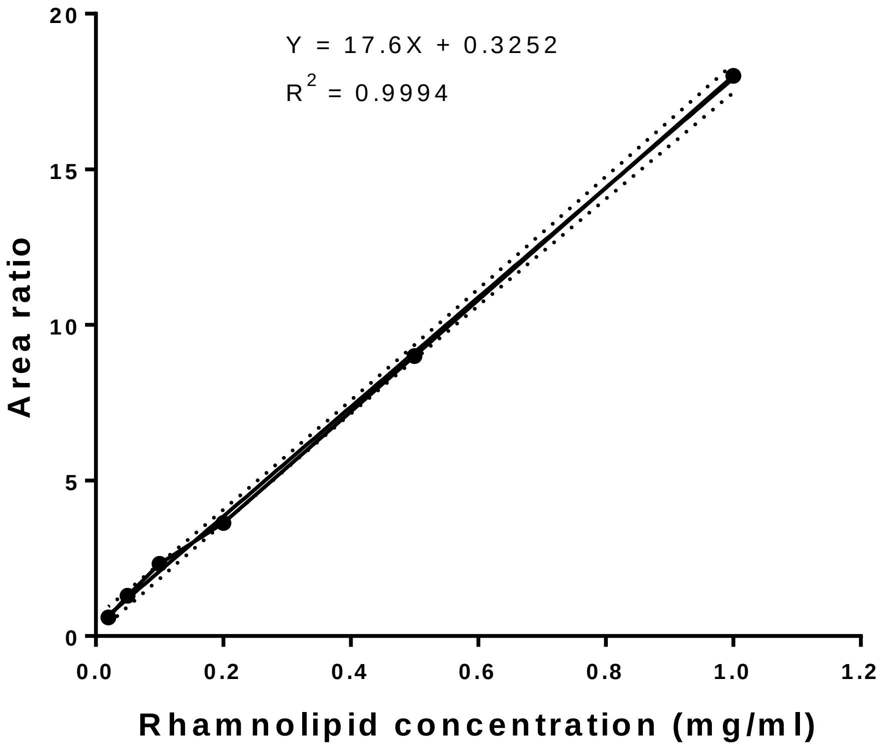

Figure S2: Quorum sensing triple mutant strains of *B. thailandensis* E264 showed reduced growth compared to single or double mutant strains and the wild type. **a** Reduction in growth of quorum sensing triple mutant strain compared to all single, double and wild type strains of *B. thailandensis* in nutrient media with 4 % (w/v) of glycerol as substrate. **b** Addition of 40% (v/v) spent nutrient broth of wild type *B. thailandensis* E264 restored growth of the triple mutant strain to similar levels as the wild type.

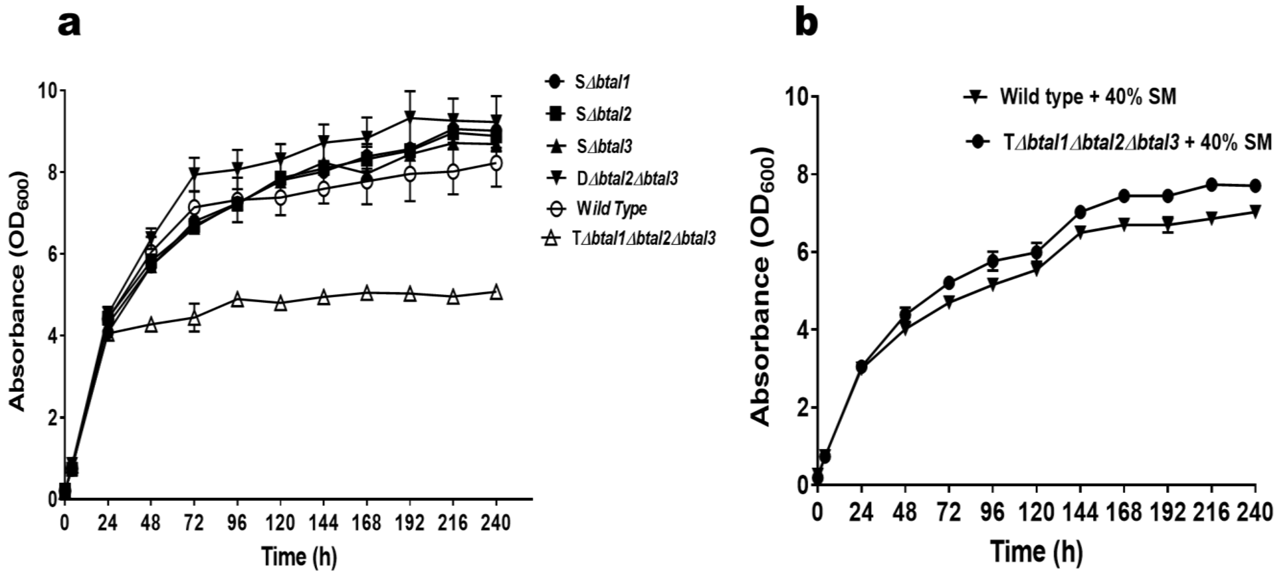

Figure S3: **a** and **b** Addition of 40% (v/v) spent nutrient broth of wild type *B. thailandensis* E264 restored surface tension and crude rhamnolipid yield respectively of the triple mutant strain to similar levels as the wild type. **c** Kaplan – Meier plot of percentage survival of *Galleria mellonella* larvae after infection with 100 cfu of wild type and triple acyl-HSLs mutant strain of *B. thailandensis* E264. Sterile phosphate buffered saline was used as a negative control in which 1 death of 30 was recorded at 30 h post infection. *n* = 30 (pooled from triplicate experiments each with 10 larvae).

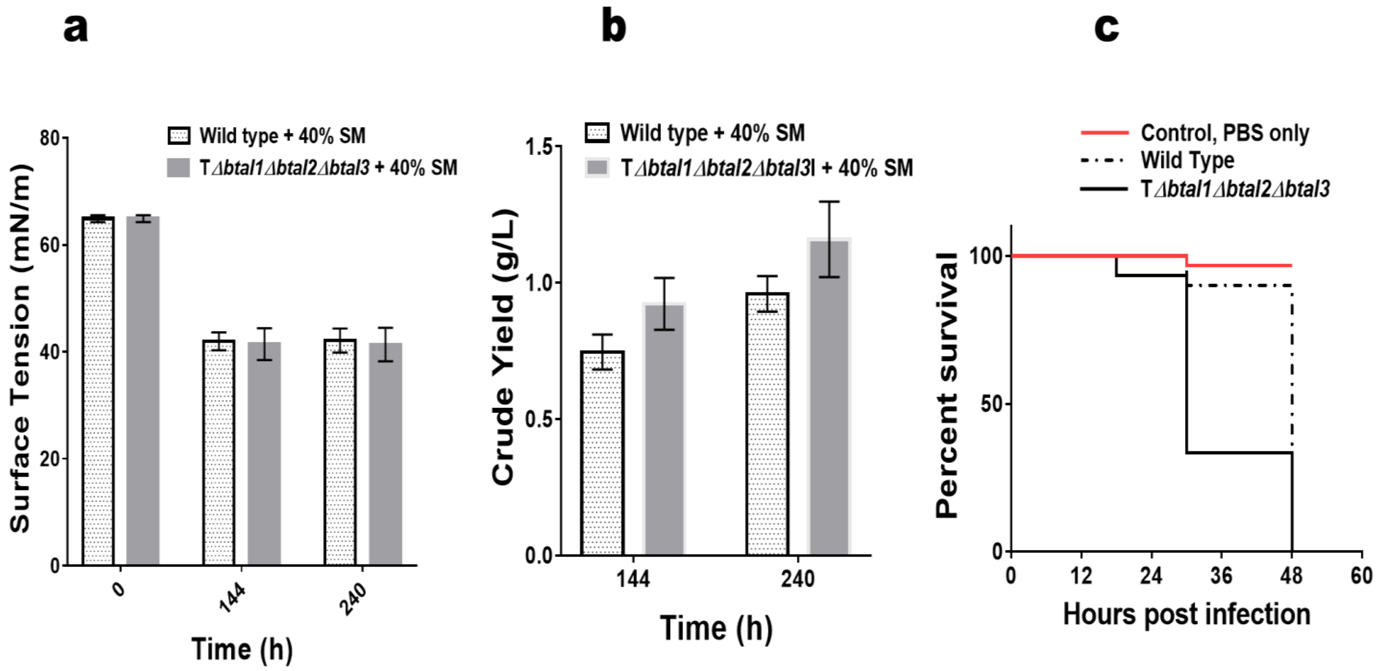

Figure S4: C NMR of PHB extracted from **a** wild type and **b** triple mutant strains of *B. thailandensis* E264.

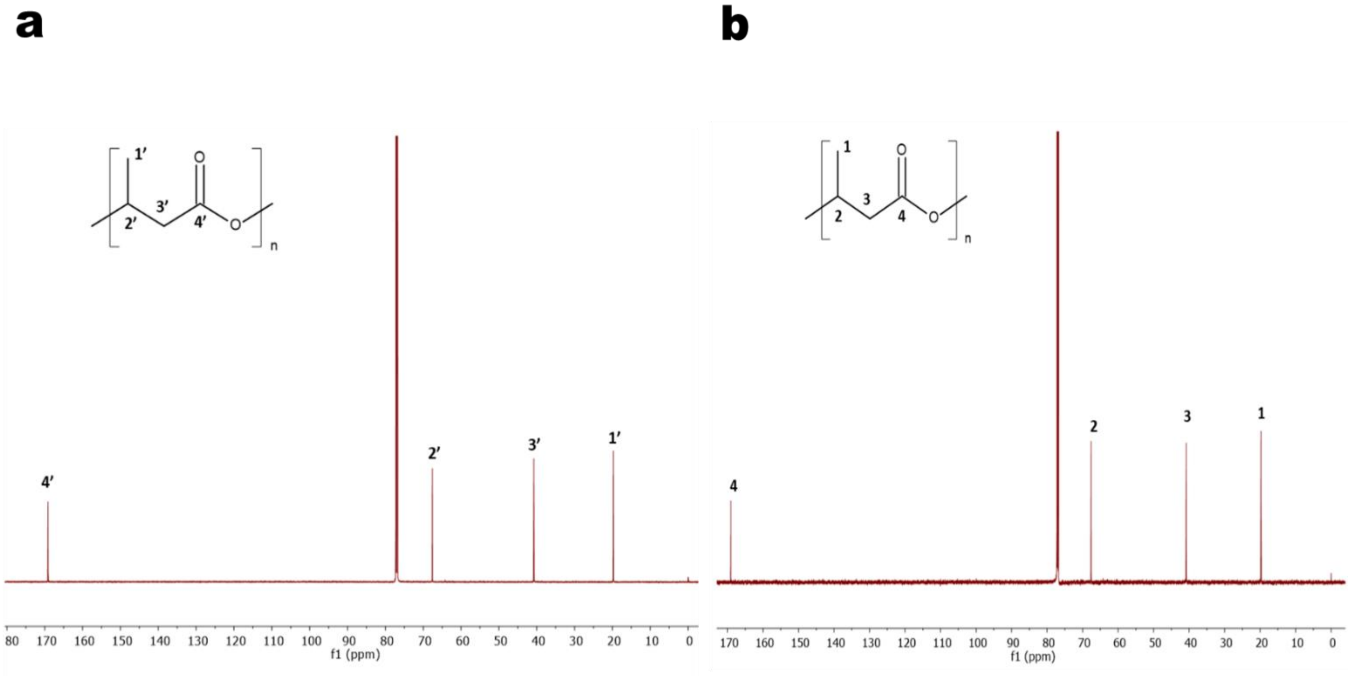

References

Brett PJ, DeShazer D, Woods DE (1998) *Burkholderia thailandensis* sp. nov., a *Burkholderia pseudomallei*-like species. Int J Syst Evol Microbiol 48(1):317-320

Chandler JR, Duerkop BA, Hinz A, West TE, Herman JP, Churchill MEA, Skerrett SJ, Greenberg EP (2009) Mutational Analysis of *Burkholderia thailandensis* Quorum Sensing and Self-Aggregation. J Bacteriol 191(19):5901-5909 doi:10.1128/jb.00591-09
